# Situation of Neuromarketing Consulting in Spain

**DOI:** 10.3389/fpsyg.2020.01854

**Published:** 2020-08-21

**Authors:** Marian Núñez-Cansado, Aurora López López, David Caldevilla Domínguez

**Affiliations:** ^1^Departamento de Historia Moderna, Contemporánea y de América, Periodismo y Comunicación Audiovisual y Publicidad, Universidad de Valladolid, Segovia, Spain; ^2^Departamento de Teorías y Análisis de la Comunicación, Universidad Complutense de Madrid, Madrid, Spain

**Keywords:** neuromarketing consulting companies, human resources, methodological and technological resources, economic resources, synchronization, interdisciplinarity

## Abstract

The latest research in Spain indicates that the most advanced neuromarketing consulting companies in the sector are those that have been able to innovate in the development of their own technologies and methodologies. Despite their reduced volume of business compared to total investment in marketing and market research in our country, there are signs that suggest these companies have great potential to improve this sector, which is still to be explored. For this reason, this research straddling the ethnographic method and the theoretical–descriptive method aims to help us better understand the characteristic features of this sector by actively listening to the professional voices that lead it. Its epistemological value lies in its contribution to understanding the business culture related to the professional development of neuromarketing in our country today. The study shows that the main human resources strategy of neuromarketing consulting companies is based on the creation of multidisciplinary work teams. In addition, most of them develop data analysis software, which they can safeguard under various types of copyright, and on other occasions they manage to patent them, to later apply them to the objectives and purposes of their company. This would also explain the widespread use of certain procedures and resources by the vast majority of the consulting companies investigated. Thus, the trend in the market is the implementation of different synchronization software to the available technological and methodological resources. Others have even gone so far as to create a new technological support to incorporate methodologies already in use. Therefore, this gives them exclusivity in their services and the necessary competitive advantage over their competitors. Among other factors, these inferences about how this sector really works are very likely to be very useful in the academic field, which will constitute a further step in generating critical thinking and in expanding the frontiers of knowledge around this discipline.

## Introduction

In recent years, neuromarketing has become a useful market research tool for small and large advertisers, who see this discipline as a new way to reach the consumer with more precision and that allows them to implement marketing strategies with better effectiveness.

Neuromarketing, as a field of study, can be defined as the application of neuroscientific methods to analyze and understand human behavior in relation to the markets and marketing exchanges (Lee et al., 2007). It is a discipline that combines knowledge of behavioral psychology, economics, and consumer neuroscience. It adapts theories and methods of neuroscience to identify neural correlations of the subject’s behavior. The primary focus of the study is the analysis of the responses of implicit processes to marketing stimuli, using neuroscience methods that make available to the researcher techniques for the study of cortical activity in terms of frequency and real time, providing a better understanding of the human brain and the cognition of the human being, eliminating possible biases that distort the research results.

It is an innovative field of research that has come to challenge the classic model of marketing research, offering better results in understanding the client’s affective and cognitive impulses, vital in decision making (Lobna Ben Nasr University of Carthage, 2014). Although, it should be considered, to be fair, that the use of psychophysiological techniques applied to the consumer research is long before the appearance of the term neuromarketing. Methodologies like pupillometry, the galvanometer, or electroencephalography (EEG; Wang and Menor, 2008) were used in the early 1970s in the study of viewers and consumers (Krugman, 1971), although the advantage of the usage of these techniques was not contemplated until the arrival of the 21st century.

The discovery of new opportunities presented by these methodologies in the field of consumer studies led to the opening of the first neuromarketing research department in the year 2002: Brighouse Neurostrategy, which in turn was a starting point for consulting companies like SalesBrain and the publication of the first scientific articles, like the one published by the research team formed by Dr. McClure, Dr. Jian, Dr. Tomlin, and Dr. Montage: *Correlates of Behavioral Preference for Culturally Familiar Drinks*, in the year 2004.

The boom in the evolution of neuromarketing consulting companies happened between 2010 and 2012, the year that culminates with the creation of Neuromarketing Business and Science Association and the birth of the Neuromarketing World Forum, the first global event that will present the advances in the new discipline. The total number of neuromarketing consulting companies has now surpassed 300 worldwide (Ulman, 2015).

Since the opening of the first neuromarketing department in collaboration with Emory University, numerous websites and posts emerged in different content platforms in which “neuro” gurus announced the panacea of marketing: the discovery of the famous buy button that represented the location in the cerebral region that kept the secret of decision-making (Senior and Lee, 2008). Many of these speakers started their careers as coaches with strong communication skills, an attractive language, and little knowledge about it, and took advantage of that to give talks under the name of neuromarketing about the skill to manage emotions in the company environment, or holding false promises with reports that served to fit infallible solutions. This type of fraud brought with it the mistrust of many businessmen and academics, raising doubts about the effectiveness of measuring techniques and its practical usefulness in the consumer research.

Nonetheless, during the first decade of the 21st century, interest in the prefix “neuro” grew, the brain sold and any discipline was likely to be analyzed under the prism of neuroscience: neurocoaching (Supriyati et al., 2016), neuroeducation (Campos, 2010), neurolaw (Amoedo-Souto, 2018), neuroaesthetics (De la Portilla, 2014), neuroanthropology (Ros Velasco, 2013), neurocommunication (Moreno López, 2018), etc. Media coverage was so high that it even caused the coining of the term *neuromania* (Tallis and Taylor, 2011), in order to reflect and define the reality that was happening about the abusive and inadequate treatment of the *neuro-* prefix, that far from entailing the mere use of the attractive of the scientific lexicon (from which incipient businesses were trying to benefit from), led to great uncertainty and mistrust around the different disciplines linked to neuroscience, seriously damaging the work of many professionals, as the mere use of a prefix does not convert a discipline into science.

Thus, we find that the substantial media coverage that neuromarketing receives is disproportionate in relation to the shortage of published scientific articles. Certainly, there is a considerable gap between research and the practice of neuromarketing, which needs to be closed through joint research proposals between scientists, academics, and professionals, which allow the creation of a neuromarketing research model (Butler, 2008). In fact, its evolution has been different from other subdisciplines in this same branch, like neuroeconomy, since it has been relegated to purely professional studies, straying away from academic interest, and being exposed to numerous criticisms from the field of ethics. Until today, neuromarketing lacks its own theoretical framework (Breiter et al., 2015).

In a content analysis of publications in specialized magazines on neuromarketing topics, only 21 out of the 66 analyzed magazines, 31.8% had articles on neuromarketing. In the research period, from 2004 to 2007, only eight articles were published in the magazines indexed in the first categories (Lim, 2018). The abuse of the prefix, the lack of a theoretical framework, which guarantees the validity of results, and the ignorance of the methodology have meant a pernicious evaluation for the discipline by the academics, which does not favor its growth or its adequate development. Analysis reveals that neurologists and specialists have higher positive perceptions about neuromarketing than academics (Eser et al., 2011). Some of these academics defend that neuromarketing is science fiction, instead of a science based on reality, given that thoughts are individual and strictly dependent on the personal experiences and personality of a person, which would make it impossible to find people with identical thoughts (Hubert, 2010) and, thus, it wouldn’t be practical implementing the results in a marketing plan.

The multiple and unjust critics received from the field of ethics have also been a tough breakdown in the correct evolution of this discipline. A fact that diverges again, with the evolution of neuroeconomic studies, whose results endorse numerous research institutions, favoring its successful theoretical and scientific development, while putting neuromarketing in check.

Another fact that is generating a large gap between academia and profession is the high cost of the neuromarketing study equipment, to which many investigators hardly have access, and the confidentiality clauses signed between consulting companies and advertisers, which has led to low cooperation (Eser et al., 2011) and, consequently, the ignorance and mistrust around these study methodologies by the academia.

The truth is that the professional reality of neuromarketing does not seem to be reflected in academic and research activity, or vice versa. We find ourselves before a new discipline, still in its infancy (Plassmann et al., 2007), that needs the cooperation between academics and specialists to build its most basic pillars and contribute to the development of a valid knowledge for society as a whole, escaping from the reductionisms and excessive simplifications that many reports show under the name of neuromarketing. Research around neuromarketing not only has to constitute a fundamental change in the way we design, promote, set prices, and package our products (Fugate, 2007), but it has to lead us to develop a solid theoretical framework that allows us to know and define this discipline.

## Objectives

This research aims to answer some initial hypotheses, that we stated as follows:

H1: The praxis of neuromarketing in Spain is carried out by multidisciplinary teams and, according to the use of a series of resources common to the group of companies dedicated to this sector, is useful in the identification of the current business model around this discipline in our country.

H2: The identification of human, technological, methodological, and economic resources used by the sector-leading neuromarketing consulting companies in our country indicates that their research is solid and is based on proven methodologies, which improve with both methodological and technical advances.

To get closer to validating or refuting the hypotheses, this research is aimed to meet the following objectives:

• Provide a real overview of the professional development of neuromarketing in our country.
• Analyze in detail the main resources of all kinds: human, technological, methodological, and economic, relative to the professional development of neuromarketing in our country.
• Establish and identify the existence of work procedures common to all leading neuromarketing consulting companies in this sector in Spain.
• Bring knowledge about the current neuromarketing praxis in Spain to academia.
• Raise the interest of professors, scientists, and professionals by establishing solvent synergies to consolidate this discipline.
• Value the professional work of this collective of experts in neuromarketing, separating it from the malpractice that prevents its recognition.

## Materials and Methods

Given the scope and complexity of the object of study, the research is structured in different phases. At first, we approach a theoretical review of the most relevant scientific literature on the proposed topic needed to contextualize the current state of neuromarketing, both from a professional and scientific perspective.

The second phase is eminently empirical character in nature and is focused on fieldwork designed to develop a rigorous database about Spanish consulting agencies. The first filter starts in Google with a word search: “neuromarketing consulting company,” which gives a total result of 15 companies related to this kind of service. To consolidate this information, we proceed to check them through a second filter, by conducting personalized interviews, one open-ended and another closed-type (in two different waves and separated in time), to the directives and communication managers in these companies, so that we could assess more adequately the real-time practice and the type of company in each case, and its degree of real affinity with the professional exercise of neuromarketing. Thus, the interviews offer valuable information that lets us discern and distinguish the different typologies of companies tied to the development of the discipline. Lastly, a third filter was implemented concerning the search for these companies in the Commercial Registry, which would provide us with accurate data about the real, labor, and financial situation of each one of them, being able to find out an even more decisive data, which is whether or not they were active. This way, from the initial 15 companies, only 6 of them could be contemplated and cataloged as neuromarketing consulting companies.

Therefore, the final sample of consulting companies currently offering neuromarketing services in our country, this being their main activity, is the following (Table 1).

**TABLE 1:** Neuromarketing consulting companies in Spain.

Consulting company	Date of incorporation	Registered office
Fusión Comunicación Empresarial	2007	Torremolinos, Málaga, Andalusia
Emo Insights Internacional S.L	2012	Madrid, Community of Madrid
Sociograph Neuromarketing S.L	2013	Palencia, Castile and León
Neurostrategy S.L	2013	Barcelona, Catalonia
Hearting The Brain. S.L Goli neuromarketing	2014	Malaga, Andalusia
Neurologycal Sciencie & marketing S.L	2015	Álava, Basque Country

*Source: Self-compiled.*

Regarding the method of selection of our sample, it should be remembered that this only contemplates businesses whose main activity is in line with the provision of consulting services in the field of neuromarketing. This specific fact justifies the dismissal of two of the companies under the *spin-off* description, originating in the university and whose activity is not truly neuromarketing, but the development of technologies and methodologies for their subsequent application in different fields of science and business (for example, security, education, etc.). Its activity focuses on R + D + i and on the sale and distribution of technological devices, hardware, and software. On the other hand, three other companies that appeared tied to this sector only carry out activities of divulging nature, like conferences, without offering studies based on the application of methodologies specific to neuroscience. In this list of non-cataloged companies, we have also discarded two companies whose main activity was the sale and rent of equipment, dedicated exclusively to the distribution. Another company was identified as an advertising agency and works with neuromarketing occasionally to add a specific value to its projects. Finally, we proved that another neuromarketing consulting company, despite having its own website and domain, only responded to the freelance work of a person that claimed to have previous experience in this sector, but it has not been proved in our country; what is far from a labor and commercial society, which is the essential condition that all listed companies must meet in the final sample under study.

In regard to the parameters under analysis, it is important noting that the research makes special emphasis on the identification, cataloging, and categorizing the different resources used by Spanish neuromarketing consulting companies in shaping their business. In this sense, discovering the type and degree of training of the people that set-up their work teams, collect precise information about the technology and methodologies used in the performance of their professional activity and understanding what is the economic magnitude of the sector and the volume of their business have earned great interest.

In a third analytical phase, we proceed to study all variables covered by the research, and a preliminary extraction of the results is carried out. Afterward, the work ends with a conclusive phase to validate or refute the starting hypotheses, and assess the degree of satisfaction of the objectives, which is achieved by observing all possible interrelations, thus leading to the establishment of the main inferences. This will aid in contemplating future research paths in this field of knowledge that could be of interest later.

Lastly, it should be noted, given the peculiarity of the research, halfway between the ethnographic method and the theoretical-descriptive method, which it draws from both primary and secondary sources. Regarding the primary sources, the open-ended interviews with the managers of the companies that are the subject of the sample stand out. As to the secondary sources, we used sources of varied nature and magnitude, such as scientific articles, and international press articles, books, websites, and information taken directly from official and institutional sources, among which the Commercial Registry stands out.

## Results and Discussion

### Human Resources

We calculated a total of 71 people employed by neuromarketing consulting companies of our country, which would suggest an average of 11.8 workers per company. However, temporary contracts are a recurring practice in two of the businesses under study, which reduced the number of permanent employees in the sector to 48.

It is very relevant that 97.1% of the total (permanent + temporary employees) have a higher university degree. Of these, only 10% have the title of Doctorate, while 25% have completed a Master’s or have completed some other kind of training in the neuroscientific field (Table 2).

**TABLE 2:** Higher qualifications obtained by members of the consulting companies.

Qualification	Total (%)
Graduate/Degree	56
Master	25
Doctorate	10
Non-regulated training	4
Vocational training	3
Postgraduate	2

*Source: Self-compiled.*

Regarding the formation of the work teams of the consulting companies by specialty, it should be noted, in the first place, that the existence of up to 23 different university degrees (Table 3) and 1 vocational training degree, particularly in the administrative field, has been determined. Of all the qualifications, we should highlight in order of predominance the ones for Psychology (13), Informatics Engineering and Business Administration (10 each), followed by Marketing and Market Research (6), continuing with the Journalism and Advertising degrees (4 each), and with a somewhat lower number Audiovisual Communication and Industrial Design Engineering (3 each). The rest of the degrees are not significant for research, since their impact responds to a more circumstantial and arbitrary criterion in each case, and it does not favor the necessary overview that lets us study this topic from a global perspective. Meanwhile, in the case of the Doctorates, Computer Science and Advertising stand out (Table 4), and concerning the Master’s degrees, the MBA specialty stands out (Table 5). Finally, it is important to mention that the multidisciplinary character of the work teams constitutes the first common distinction for our whole studied sample. So, this circumstance supposes a fundamental bias in the research, bringing a conclusive item.

**TABLE 3:** Representation of university degrees by specialty in consultancy employees.

Degree Courses	%
Psychology	16.7
ADE	12.8
Computer Engineering	12.8
Marketing and market research	7.7
Journalism	5.1
Advertising	5.1
Sociology	5.1
Audiovisual Communication	3.8
Industrial Design Engineering	3.8
Business	2.6
Statistics	2.6
Mathematics	2.6
Law	2.6
Electronic Engineering	2.6
Telecommunications Engineering	2.6
German Philology	2.6
Graphic Design	1.3
Neurology	1.3
Industrial Engineer	1.3
Engineering in Oceanographic Sciences	1.3
Chemistry	1.3
English philology	1.3
Teaching	1.3

*Source: Self-compiled.*

**TABLE 4:** Doctorate specialties.

Titulaciones Superiores	%
Ph.D. in Computer Science	29
Ph.D. in Advertising	14
Ph.D. in Marketing	14
Ph.D. in Mathematics	14
Doctor of Neuroscience	14
Doctorate in Marine Ecology	14
Post-doctorate in Artificial Vision	14

*Source: Self-compiled.*

**TABLE 5:** Specialties of higher degrees.

Specialty of Higher Degrees	%
MBA	24
Private and non-regulated training in Neuroscience	14
Master’s in Retail	9
Master’s in Neuromarketing	5
Master’s in Marketing	5
Master’s in Neuropsychology	5

*Source: Self-compiled.*

By neuromarketing consulting companies, Fusión Comunicación is the one that presents a higher percentage of employees with Master’s degrees, specifically in the Neuromarketing specialty, with up to 7 people of a total of 12, whose university training is, also, Administration and Direction of Businesses, that is, the second university degree with the most representative percentage among the profiles used by the sector (Table 6).

**TABLE 6:** Combined table of data by consultant.

Consulting company	Methodologies	Own methodologies	Human Resources
Sociograph	GSR	Sociograph	3 Journalism/1 Marketing/1 Statistics
	Eye tracker Mobile		1 Advertising (Dr.)/1 English Philology/
	Facial Coding		2 Business
	EEG		
	Beacons		
Emo Insights	GSR	FEM Methodology	1 Marketing (Dr)/1 ADE/Law
	Google Glasses		1. Mathematics/1 Statistics/1 Chemistry/MBA/1 Industrial engineer/neuropsychology
	EEG		2 Neuropsychology/(MSC) Neuromarketing/
			1 Sociologist/1 Psychologist/1 Graphic designer/1 Law/Communication
			1 Journalism/1 Computer Science
Neurostrategy	VPA	NEUROMAP	1 Mathematician + Doctorate + MBA/4 Psychology/1 Industrial engineer/2 Industrial Design Engineering/2 Computer engineer + Doctorate + MBA/2 Computer engineers/2 Engineering
	Heatmaps		1 Telecommunications/1 German Philology
	EEG		
	Eye tracker Mobile		
Neurologyca	Eye tracker VR	Multisource	1 Marketing/German Philology/Teaching/Neuroscience Course. 1 Marketing/Neuroscience Course/1 ADE/MBA/1 ADE/MBA/Neuroscience Course
	Eye Mobile	Neurofan	1 Psychologist (Dr. in Neuroscience)/2 Sociology/1 Engineering in Sciences/Oceanography (Dr. in Marine Ecology)/Post-doctorate in Artificial Vision/1 Computer Science
	Facial coding	Neurotaste	
	EEG		
	GSR		
	Cardio biosensors		
	Pupillometer		
	Thermograph		
Goli Neuromarketing	Pulsometer	Goli Neuronline	1 Computer Specialist + Psychologist + Master’s in Marketing + Master’s in Neuropsychology/2 Computer Specialist/1 Neurology/2 Marketing (MSC in Retail)/2 Psychology/2 FP Administration
	GSR	Golihelmet	
	EEG	Goli Touchsense	
	Eye tracker Mobile		
Fusión Comunicación	Eye tracker Mobile	Fusionlab	7 ADE/(MSC) Neuromarketing/
	Eye tracker Fixed		1 Sociology/1 Psychology/2 Advertising and Audiovisual Communication/1 Computer engineer/Advertising/
	GSR		
	Facial coding		
	EEG		

*Source: Self-compiled.*

The consulting company with the most number of Ph.D. employees would be Neurostrategy, with three doctors, as well as two other employees with an MBA, but we have to keep in mind that the work teams of this company are variable, since the employees and their profiles are selected for hiring based on the service they are going to provide at all times. Therefore, seen in this way, it corresponds to Neurologyca to occupy the second place according to the criterion of the higher level in training of its work team since it is about a team of nine permanent employees, of which two are doctors (one in the field of Neuroscience), two have an MBA degree, and two others also have private training in Neuroscience, which places Neurologyca with a total of six employees with higher education. As a curiosity, Neurologyca stands out from the rest for counting on an academic profile uncommon in this professional field, like Engineering in Oceanographic Sciences, which one of its doctors possesses, with a post-doctorate in Artificial Vision (Table 6).

Regarding the number of employees that certify the dominant degree, in Psychology, the consulting companies Goli Neuromarketing and EMO Insights top the ranking in the most hired with that profile, with three employees each. However, sometimes EMO Insights expands that number to four because another one of the members of its indirect team is a psychologist as well. Meanwhile, Neurostrategy claims to have four psychologists as well, always on demand and depending on the necessities of each project, which gives a temporary character to the hiring of these profiles. Thus, even if the data are more significant because they prove that degrees in psychology are the most valued and requested by the company, it is not feasible placing it as #1 in the ranking of companies with the most number of employees with this profile, given that it is a more unstable work character. Meanwhile, Sociograph is the only consulting company that does not have any psychologist in its workforce.

On the other hand, if Fusión Comunicación is the consulting company that concentrates a higher percentage of employees with an academic profile in Business Administration and Management, in the case of the computer science degree, equally representative in the composition of the teams, Goli Neuromarketing is the company with the highest number of qualified employees in this knowledge field in the sector, with three computer engineers. Likewise, Goli Neuromarketing shares with Fusión Comunicación the fact that it does not have any doctorate among its employees. It is followed by Neurostrategy with two computer scientists, but again, the data have to be taken very cautiously, because, as we know, the hiring of employees by this consultancy is variable and responds to a criterion of consulting needs according to the type of projects.

### Methodological and Technological Resources

To be able to achieve this overview in the study of the methodologies used by our country’s neuromarketing consulting companies, it has been clearly decisive to distinguish between own methodologies and third-party methodologies (Table 6). In this sense, it is understood by its own methodology, the one that the company has developed by its own initiative and based on an exclusive software, to fulfill specific objectives. This is important for them to be able to acquire any specialization in their sector and the necessary competitive advantage over the rest of the companies. On the contrary, third-party methodologies are the ones that the neuromarketing companies use in their studies through the acquisition of licenses because they have not been developed by them in any way, but their validity is unavoidable and their usage appears indispensable for the adequate provision of their services. In that case, the most frequently used third-party methodologies come from technological equipment and software developers specific to neuromarketing, like iMotions and Bitbrain, the main supplier in the sector in Spain. In the case at hand, it is noted, in general for the entire sample, that the *modus operandi* of the development of in-house methodologies often concludes either with a patent or by registering them through copyright, something they use strategically to add value to their business. On numerous occasions, the methodologies developed in-house are a redesign or an adaptation of third-party methodologies. Both in-house and third party are necessary, while in-house methodologies and technological implementations enrich their research, helping them in orientating and extracting data with more precision according to the specific objectives of each project; third-party methodologies are the vital support from which the companies start any of their studies.

For this reason, it is possible to explain that, throughout this research, we have met consulting companies that claimed to have actively participated in the creation of some of these third-party methodologies. Thus, from the deficiencies found in the praxis and the rise of new necessities in their research, almost permanently, while their businesses grow, they demand improved methodologies impossible to develop in-house, which leads them to this situation of *ad hoc* collaborators of the main developing companies of technology and methodologies aimed at neuromarketing. The fact that the implementation of upgrades in the sector begins and ends exclusively in the business field, without any connection with Academia (Nuñez-Cansado et al., 2018), proves the serious issues it faces to grow with higher chances of success, and become a discipline defined on the basis of solid scientific principles. In this sense, the establishment of synergies between professionals and academics is a top priority matter to achieve its consolidation and earn a place in the broadening of the borders of knowledge.

On the other hand, we were able to detect a mechanism common to all companies regarding the development of their research, where the implementation of multiple methodologies synchronously and in an integrated way is constant. This consistent way to proceed counts with the full approval of the group of analyzed companies, and its establishment, although we were told that it was very recent, has been consolidated rapidly in the sector as the applicants for these types of services became more demanding. In this way, the data collection protocol that needed to use of one or two methodologies independently was left behind, and only in some cases, they were simultaneous, to give way to new forms that respond to the application of software capable of integrating multiple methodologies, which allow the synchronization of the collected data and the creation of behavioral patterns (Table 7) in combination with other more universal methodologies, such as those provided by iMotions. Each of the consulting companies of our sample has its own methodologies or technological advances adapted to the basic methodologies, which gives them uniqueness and reverses a clear brand differentiation.

**TABLE 7:** Own methodologies.

Consulting Company	Methodologies
Sociograph	Sociograph
Emo Insights	FEM Methodology
Neurostrategy	Neuromap
Neurologyca	Multisource
	Neurofan
	Neurotaste
Goli Neuromarketing	Goli Neuronline
	Golihelmet
	Goli Touchsense
Fusión Comunicación	Fusionlab

*Source: Self-compiled.*

According to these advances, the common methodological process is, therefore, the synchronization of, at least, two measurement methods, although this number is often higher. This puts us before another item of high magnitude for research, because of how much it contributes to the identification of the methodological markers that could be key in the description of a scientific method par excellence for neuromarketing.

**FIGURE 1:**
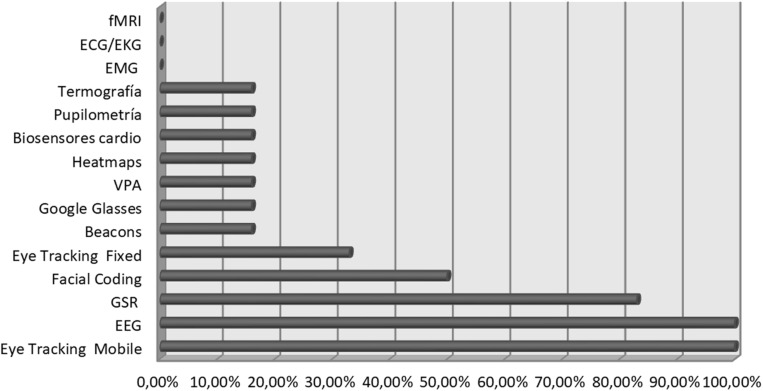
Consulting companies methodologies.

The neuroscientific methods are made up by the use of tools and techniques to register neural and cerebral activity in real-time, during the behavior of the subject. One hundred percent of the companies rely on methods of Eye tracker Mobile, EEG, and GSR, being the most used (Figure 1). The use of these tools happens, assiduously, through software that allows the integration and synchronization of all of them at the same time, which is beneficial by allowing the collection of data of varied nature, from measurement procedures completely different and complementary.

Half of the sample uses Facial Coding methods in its studies, and only 33% of them use a fixed technology like Eye tracker. Techniques of Beacons, Heatmaps, cardio biosensors, pupilometries, and thermographies are used to a lesser extent, only by 16% of the companies. Lastly, none of them count with EMG, ECG/EKG, or fMRI techniques (Figure 1).

The fMRI technique presents itself, according to experts, as the most precise measurement. Lim (2018) pointed out that it had been used in up to 42% of the scientific research done in this field. This methodology allows you to measure and map the cerebral activity through changes associated with blood flow detected by a magnetic resonance scanner, with a high spatial resolution and a low temporal resolution. Even though it allows analyzing the purchase intent, activation of pleasure, emotional arousal, decision-making processes, or degrees of concentration, among other things, the research tendency in the neuromarketing field implies great mobility of the used devices, which in this case is non-viable and relegates the use of the fMRI to specialized research centers. Likewise, its high cost is the main access barrier to this technology for these microbusinesses, just like it is derived from the financial results of the analysis, which is why no company uses the fMRI technique.

Electroencephalography and the Eye tracker Mobile are the two most used methodologies in the research of consulting companies, usually in a synchronous manner. Electroencephalography records electrical brain activity using biometric sensors. In the medical field, wet sensors are often used, but although their use is more reliable, they are impractical in a real marketing environment. The consultancies have dry sensors in helmets or headsets, with Bluetooth that allow the necessary mobility. This is a technology that lets you detect brainwaves produced in response to stimuli, and it has a high temporal resolution and a very low spatial resolution compared to fMRI. It allows the measurement of the attention stages, emotional, arousal, and the levels of cognitive processing or purchase intent.

All consulting companies carry out their studies supported by Eye tracker Mobile, with just over 30% of them using Fixed Eye tracker. This technology records the positions and movements of the eyes in response to stimuli that allow a better understanding of the attention process and the creation of fixation patterns. It has a high temporal resolution and very low cost. The quality of the results is largely tied to the characteristics of the equipment. Most consulting companies have 60-Hz equipment, which lets them make very reliable fixation maps. This is a valuable tool that complements EEG and GSR, and it really allows them to explain which stimulus is really causing the response obtained with other methodologies.

The third most used methodology is the galvanometer or GSR. This method records slight changes that are produced by the activation of the autonomous system. The measurements vary according to the system used, and both the recording of resistance and electrical conductance of the skin can be valid. It has a low temporal resolution because there is a gap between the measurement of the response and the activation. It can only measure the degree of excitement, and in no case can it measure the emotional valences. Using this tool without synchronizing it with other methods like facial coding can give way to reductionist and simplified conclusions, which is why the consulting companies should use it in synchronization with the facial recognition methodology. Despite this, two of the companies do not use GSR synchronized with facial recognition. This circumstance would be a great obstacle if the objective of the research was to show the valences and intensity of the emotions.

The fourth most used methodology is facial recognition. It is a supporting method that contributes with the valences and the affective intensity of the emotions. The methodologies used are varied. The technology that supports facial recognition consists of a camera that recognizes muscle movement and a probabilistic approach analysis software that concludes in an emotional diagnosis. The consulting companies use it synchronously with GSR or EEG. The technology used in this methodology allows them to show from 4 to 16 different emotions with different intensities, so a study done with one or the other technology may vary substantially.

Regarding this resource set, this study has served to bring to light that neuromarketing consulting companies do not value the technological differences that they have for the realization of their studies, focusing all the interest in the usage of in-house methodologies. This shows several shortcomings, the first one being the lack of the commercial vision needed to make their business grow, ignoring this argument against all odds and sense, when ignoring the differences between, for example, when using an Eyetracker of 30 or 60 Hz in a measurement, the quality of results obtained vary completely. Secondly, this could mean that they do not truly know their competition and their available resources, when they cannot find in their technological differences, a fulcrum from which they can self-define and differentiate themselves from the rest. If we add to this, that most try to do by giving a heavier weight to the cache of the brands they work with, but being tied to confidentiality contracts they barely can refer to those facts to hold the prestige they should, we can conclude that the consulting companies should rethink the generation of their own business formulas based on the validity of their methodologies, and even, in the near future, give way to strategic advertising and communication plans to strengthen and expand their businesses.

In general terms, the practice of neuromarketing presented by the consulting companies implies, even for them, a continuous learning experience. This way, even though most of them acquired or rented third-party methodological and technological resources, as is the case with the most widely used ones—EEG, Eye tracker mobile, and GSR (Figure 1)—sometimes projects arise from them for which these are insufficient, data collection from one methodology is not valid enough, and they need to develop in-house methods, methodological or technological, according to the needs of each job they face. This, at the same time, generates very important feedback for the R + D + i companies that lead the market for the methodological and technological development of these resources, which is so necessary for them, and in whose ideation and design they frequently collaborate, moved by the interest of improving in their profession and offering quality services, which are increasingly more rigorous. However, sometimes ingenuity arises from the necessity and some create their technological support, like the Goli Helmet patent from Goli Neuromarketing, or Sociograph’s electrodermal activity (EDA), also a patented technique that takes EDA as a reference, although as a group response, not an individual one. In this sense, it is an original and specific contribution, based on a patent, which is used exclusively as a methodology by only one of the consultancy firms studied (Martínez-Herrador, 2003; Martínez-Herrador et al., 2020).

### Economic Resources

**FIGURE 2:**
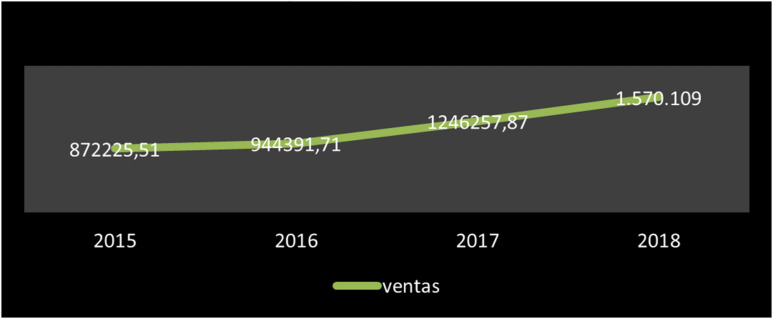
Sales development (Sales value in EUR).

The consulting companies under study are classified as micro-enterprises with private capital from the third sector at the national level. The volume of their sales is always under 2 million euros. It is a market on the rise, with three very solvent companies, that show a very consolidated economic activity. The sales evolution in consulting companies is increasing (Figure 2). Also, all the consulting companies, except one, show increase in their sales. The net result is very variable between them (Table 8). In 2018, the total number of sales was of €1,570,109. Only 33% of our sample showed negative net results in that year.

**TABLE 8:** Economic resources: Commercial Registry.

Consulting company	Net income 2018	Sales			
		2015	2016	2017	2018
Emo Insights Internacional S.L	2935€	403,715.16€	335,452.50€	439,669.28€	**472,302€**
Sociograph Neuromarketing S.L	40,875€	131,715€	216,671.44€	317,081.64€	**407,856€**
Neurologyca Sciencie & marketing S.L	38,426€	96,553.34€	193,288.30€	270,307.46€	**415,862€**
Neurostrategy S.L	−1856€	194,704.60€	162,180.38€	164,921.89€	**256,276€**
Fusión Comunicación Empresarial S.L	−16,279€	40,543.42€	31,366.75€	34,345.80€	**17,813€**
Hearting The Brain. S.L Goli neuromarketing	1545€	4,993.99€	5,432.34€	19,931.80€	**21,881€**

*Source: Self compiled.*

As consulting companies, taking account specifically of their sales volume, stand out in order, for the last year of recorded economic activity (2018), EMO Insights, Neurologyca, and Sociograph all exceed €400,000. Although these data help us get an idea of who exercises current leadership over the sector, an earlier study already served to detail all financial data that were known until now regarding the development of professional activity in neuromarketing in our country (Nuñez-Cansado et al., 2019).

Despite it being a market sector controlled by a small number of companies, in general terms, the sector shows strong growth, doubling its sales volume in four consecutive years. This is very important if we remember that none of these companies are more than 13 years old.

## Conclusion

The neuromarketing sector presents itself as an incipient field with a low net result, although with a notably growing indicator in the sales figures, that has exponentially increased to double in the last 4 years. Its total turnover amounts to 1570.09 euros, which makes its economic contribution very residual for the marketing field, according to the data given in the report made by AMES (2018), whose total revenue corresponds to the figure of 31,794 million. Thus, the neuromarketing field represents barely 4.9% of the total revenue, which could be explained by the low volume of businesses in the field.

In line with these circumstances, the contributions of the ethnographic method of study are highly interesting, and it should be remembered that all directives of the sample noted in their interviews that the malpractice by unprofessional companies in our country had as a consequence the rise of a scenario of disbelief and bad press around neuromarketing, which made its development difficult until today. Nonetheless, the Latin American markets ignored the negative trends of Spain, showing a higher permeability in the hiring of these types of services. Consequently, some of the companies in our sample participated actively in Latin America carrying out specific jobs in the field of neuromarketing consulting.

The professional exercise of neuromarketing requires integral experts (Crespo-Pereira et al., 2016), and certainly the works teams are interdisciplinary. Inadequate professional competence could give way to under- or overestimating neuroscientific findings and misreporting advances in the findings, which could lead to faulty planning and strategy contemplation (Plassmann et al., 2012). In this sense, the analysis of degrees shows a great variety of academic profiles that complement each other, but in other cases, profiles that do not have enough knowledge from which to adequately implement the results and formulate the necessary questions for each technique. A recurring fact throughout the research manifests the existence of redundant confusion on the part of professionals regarding the concepts of “technology” and “methodology.” Mainly, the inadequate observed descriptions regarding their use impeded the exact determination of their etiology. This difficulty in knowing exactly what they are researching with and the reason of it is not accidental and was raised in an earlier study (Fisher et al., 2010).

However, the study reveals data relating to the valuable methodological and technological resources, since it manifests for the first time the existence of common and shared procedures in practice regularly, which may constitute a base for the neuromarketing research model, revealing that the Eye tracker, the EEG, and the GSR (Figure 1) are the most recurrent methods. Also, the application of multiple methodologies in a synchronized way has become the dominant tone in the marketplace studies realized in this field, having contributed to improving the quality of the services offered by the consulting companies and boosted its effectiveness. The collection of data using only one method does not appear to be an adequate practice; the synchronization and the creation of pattern analysis models can make a clear difference between neuromarketing consulting companies.

Besides the most widely used methodologies, all consulting companies implement others to complement their studies by differentiating from the competition. This is how they contribute their added value and place themselves in a more advantageous position in the market, keeping the methodologies or algorithms used to get their reports privately. The most striking thing in this sense is how this study has contributed to the conclusion that all the sector-leading consulting companies in Spain have maintained their activity over time, overcoming the difficulties that have emerged, thanks also to their work as their methodology and technology developers (Table 7). Their growth and sustainability would not have been possible without the performance of this important work.

In this sense, this study has brought to light that neuromarketing consulting companies do not value their technological differences and their methodological procedures, despite the work done in both fields. This makes us think that while offering their services to clients, they do not emphasize on these notorious circumstances. On the other hand, this reality could reflect a lack of knowledge about the competition. In any case, before a situation like this, it is recommended that the consulting companies revise their commercial policies and act accordingly to create more effective communication plans in the future.

Lastly, the interrelation of the parameters studied does not make it possible to establish useful criteria on which to highlight the importance of some companies over others. Generally, all companies show great capacity of adapting to the market’s needs, as well as finding and recognizing business niches to serve with their studies. The idiosyncrasies particular to each of them constitute great value as a whole. This way, even when they work with similar and identical methodologies for similar or identical sectors (telephony, retail, food, etc.), each company maintains a *savoire-faire*, which they keep a secret.

Undoubtedly, any business tries to shield itself from the competition, become indispensable, and grow. To do that, discretion and prudence are always good allies, but in the case of neuromarketing consulting companies, they are not enough to consolidate their sector, that despite being on the rise, is full of opportunities. To continue cooperating, collaborating, and generating feedback among academics, scientists, and professionals is the infallible recipe to follow in order to save this discipline from the wreck in which it would otherwise be immersed.

## Data Availability Statement

The raw data supporting the conclusions of this article will be made available by the authors, without undue reservation.

## Author Contributions

MN-C: theoretical framework, data analysis, discussión and conclusion. AL: methodology, data analysis and conclusion. DC: bibliographic revision. All authors contributed to the article and approved the submitted version.

## Conflict of Interest

The authors declare that the research was conducted in the absence of any commercial or financial relationships that could be construed as a potential conflict of interest.
